# Lifestyle interventions for type 2 diabetes prevention in women with prior gestational diabetes: A systematic review and meta-analysis of behavioural, anthropometric and metabolic outcomes^☆^

**DOI:** 10.1016/j.pmedr.2015.05.009

**Published:** 2015-05-24

**Authors:** A.S. Gilinsky, A.F. Kirk, A.R. Hughes, R.S. Lindsay

**Affiliations:** a School of Psychological & Health Sciences, University of Strathclyde, Glasgow, Scotland, G1 1QE, United Kingdom; b British Heart Foundation Glasgow Cardiovascular Research Centre, 126 University Place, Glasgow G11 8TA, United Kingdom

**Keywords:** Gestational diabetes, Lifestyle, Review

## Abstract

**Purpose:**

To systematically review lifestyle interventions for women with prior Gestational Diabetes Mellitus (GDM) to report study characteristics, intervention design and study quality and explore changes in 1) diet, physical activity and sedentary behaviour; 2) anthropometric outcomes and; 3) glycaemic control and diabetes risk.

**Methods:**

Databases (Web of Science, CCRCT, EMBASE and Science DIRECT) were searched (1980 to April 2014) using keywords for controlled or pre–post design trials of lifestyle intervention targeting women with previous GDM reporting at least one behavioural, anthropometric or diabetes outcome. Selected studies were narratively synthesized with anthropometric and glycaemic outcomes synthesized using meta-analysis.

**Results:**

Three of 13 included studies were rated as low bias risk. Recruitment rates were poor but study retention good. Six of 11 studies reporting on physical activity reported favourable intervention effects. All six studies reporting on diet reported favourable intervention effects. In meta-analysis, significant weight-loss was attributable to one Chinese population study (WMD = − 1.06 kg (95% CI = − 1.68, − 0.44)). Lifestyle interventions did not change fasting blood glucose (WMD = − 0.05 mmol/L, 95% CI = − 0.21, 0.11) or type 2 diabetes risk.

**Conclusions:**

Lack of methodologically robust trials gives limited evidence for the success of lifestyle interventions in women with prior GDM. Recruitment into trials is challenging.

## Highlights

• There is good evidence for diet change after lifestyle intervention in GDM.
• Limited evidence of PA, anthropometric or glycaemic change with lifestyle in GDM
• More methodologically robust trials of lifestyle intervention in GDM are required.
• Recruitment to lifestyle intervention trials for women with GDM is challenging.

## Introduction

Gestational Diabetes Mellitus (GDM) is a form of diabetes that is diagnosed during pregnancy and affects up to 16% of pregnant women (Coustan et al., 2010). Recent changes in guidelines (Coustan et al., 2010) for clinical diagnosis of GDM, in addition to upward trends in obesity and unhealthy lifestyles, has increased the number of women being diagnosed (Dabelea et al., 2005). Progression to type 2 diabetes for women with GDM is reported to be between 15 and 50% at 5 years (Kim et al., 2002). Furthermore weight and BMI are significant predictors of development of type 2 diabetes at 15-year follow-up (Linne et al., 2002).

Guidelines on type 2 diabetes prevention (National Institute of Health and Care Excellence, 2008) clearly state that high-risk populations, such as women with GDM, should be offered lifestyle interventions. In women with GDM, physical activity and dietary change successfully improves glycaemic control, body composition, reduces requirements for insulin and may prevent onset GDM in subsequent pregnancies and future development of type 2 diabetes (Ruchat and Mottola, 2013, Bao et al., 2014). The Diabetes Prevention Program (DPP) showed that lifestyle interventions and Metformin reduced type 2 diabetes incidence by 58% and 31% respectively in people with impaired glucose tolerance (IGT), including those with a history of GDM (Ratner et al., 2008). These reductions in incidence rate were maintained up to 10 years (Knowler et al., 2009).

Several studies examining the effectiveness of lifestyle interventions in women with prior GDM have recently been published (Cheung et al., 2011, Ferrara et al., 2011, McIntyre et al., 2012) and more trials are in progress (Ferrara et al., 2014, Infanti et al., 2013a, Shih et al., 2013), however, evidence from intervention trials within the general population of pregnant and postpartum women suggests that behaviour change is challenging in these groups (Currie et al., 2013, Gilinsky et al., 2014/07). Similarly, research with GDM populations have reported difficulties recruiting or retaining participants (Cheung et al., 2011), and compared with women with IGT and no prior history of GDM, poorer engagement in lifestyle changes (Ratner et al., 2008). These findings suggest that lifestyle interventions and research methods may require adaptation for women with GDM. Lifestyle interventions for preventing type 2 diabetes in women with prior GDM have not been systematically reviewed to date, yet this is important to inform future research and practice.

The objectives of this research were to systematically review published studies investigating lifestyle interventions for women with previous diagnosis of GDM to explore changes in 1) behavioural outcomes (diet, physical activity and sedentary behaviour); 2) anthropometric outcomes and; 3) glycaemic control and diabetes risk. Study characteristics and quality in addition to intervention content and design are also reported.

## Methods

The review was registered with PROSPERO International prospective register of systematic reviews (www.crd.york.ac.uk/PROSPERO). Methods of the review followed COCHRANE (http://www.cochrane.org) and PRISMA guidance (http://www.prisma-statement.org), which specify recommended quality criteria for conducting and reporting systematic reviews and meta-analyses.

### Study selection

We included lifestyle intervention studies targeting women with previous diagnosis of GDM. Although recruitment and interventions could commence during pregnancy, as the focus was on prevention of type 2 diabetes in women with prior GDM, studies were only included if they reported interventions and outcomes during the postpartum period. Included interventions were those promoting weight loss or physical activity, change in diet, or decreasing sedentary behaviour and delivered via structured exercise programmes, lifestyle counselling, health education, and self-management programmes. Studies had to include at least one behavioural (diet, physical activity or sedentary behaviour) anthropometric (weight, BMI, percent body fat, waist or hip circumference) or diabetes outcome (measure of glycaemic control or diabetes risk). We included randomised controlled trials (RCTs), controlled trials or pre–post studies in the systematic review, however only RCTs were included in meta-analysis. We included all control/comparison groups (e.g. usual care, a waiting list, no treatment and/or a minimal intervention (e.g. leaflet)).

Studies not in the English language; dissertations, expert opinion, non-published studies and conference abstracts were excluded, however we contacted authors of relevant conference abstracts/protocol/baseline/methods papers to identify published data. Studies conducted with pregnant women with no diagnosis of GDM, pre-existing or current type 1 or type 2 diabetes, or women with a positive glucose challenge test who did not meet criteria for GDM were also excluded. There were no exclusions based on time since GDM diagnosis.

**Fig. 1:**
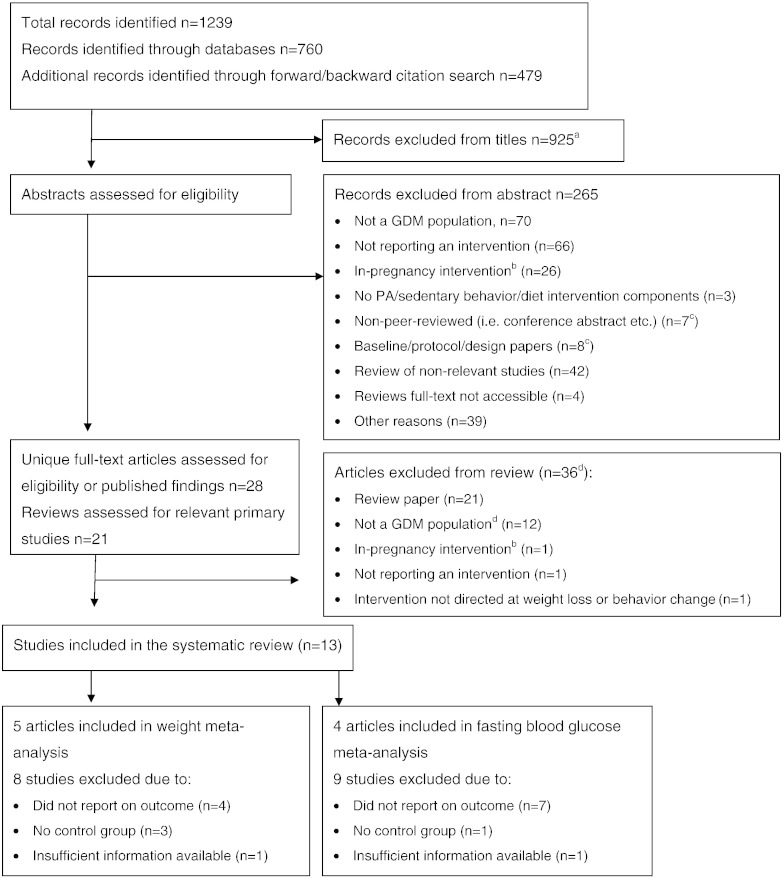
Flow of search and selection process. Notes. ^a^May include duplicates of some records identified though the cited reference search had already been considered following the database search. ^b^In-pregnancy interventions were excluded if the outcomes were reported in-pregnancy only. We considered interventions that recruited/began in pregnancy if the outcomes were postpartum. ^c^14 authors were contacted to check for peer-reviewed published manuscripts. Seven did not respond, four advised no published information was available, two linked us to papers already identified and one provided a new paper. ^d^Four authors were contacted to request information regarding the subset of GDM women from their sample and five to request further information for completeness of data extraction. Information was not provided in six cases, resulting in four articles being excluded from the review.

Studies obviously not meeting inclusion criteria were eliminated at title stage, thereafter abstracts were reviewed. Fig. 1 notes reasons for exclusion. Remaining studies were downloaded for full-text review.

### Data sources and searches

The search strategy was developed in consultation with a subject specialist librarian. We searched the following databases: Web of Science (inclusive of Medline), Cochrane Library: Cochrane Central Register of Controlled Trials (CCRCT), EMBASE (on OVID), Science DIRECT from 1980–April 2014, selecting English-only abstracts. Terms used were: (pregnancy diabetes mellitus or gestational diabetes) AND TOPIC: (intervention* or prevent*) AND TOPIC: (“physical activity” or walking or exercise or sedentary or sitting or diet or lifestyle) AND TOPIC: (controlled study or trial*). Reference lists from all included papers were searched.

### Data extraction and quality assessment

One author searched and extracted data from all studies (ASG). Two authors (AFK & ARH) reviewed in total 50% (i.e. 25% each) of full-text studies to check they met inclusion criteria, check correct extraction of data and assess quality assessment indicators. A data extraction form was developed to extract data on: study population, interventions and comparator conditions, recruitment and retention methods and all relevant outcomes (i.e. behavioural, anthropometric, progression to type 2 diabetes and glycaemic control). The CONSORT flow diagram was used to extract numbers approached, randomised, allocated and receiving the intervention/comparator conditions and numbers and reasons for loss-to-follow-up (Moher et al., 2001). Authors were contacted if further information was required.

Methodological quality was assessed using criteria for judging bias in intervention studies recommended by Cochrane. All studies were coded as adequate, not adequate, unclear or not applicable in relation to sequence generation, allocation concealment, blinding of outcome assessors, retention at follow-up and handling of data (criteria for coding given in Table 2). These quality indicators were then used to assign each study with an overall risk of bias rating of high, low or unclear.

### Data synthesis and analysis

All extracted study characteristics and risk of bias data was entered into evidence tables (See Table 1, Table 2). A synthesis is summarized in the results section below. After extraction the following outcomes were synthesized using meta-analysis: anthropometric – change in weight (available in five studies) and glycaemic – change in fasting blood glucose (available in four studies). Inclusion within the meta-analysis was dependent on the study being of a randomised controlled design and data being reported within the paper or from author contacts. We did not conduct a meta-analysis of behavioural outcomes due to large variability in the methods of measurement and units of measure for the behaviour.

For the meta-analyses, we conducted random effects analysis in RevMan 5.0, analysing the between-groups difference in each outcome at the last follow-up point or between groups change from baseline (depending on what was reported in the published paper) using the weighted mean difference (WMD) measure. We present outcomes in terms of efficacy in the short-term (e.g. 13 weeks or less follow-up), short-medium term (i.e. 6 months follow-up), medium-term (i.e. 12 months follow-up) and long-term (i.e. 24 months follow-up or greater). Heterogeneity was investigated using chi-square (Q-statistic), based on observing a p-value of < 0.05, and the I2 test, with levels > 50% suggestive of substantial heterogeneity. We did not conduct assessment of publication bias due to the small number of studies eligible for inclusion in the meta-analysis.

## Results

### Identification of studies

A total of 1239 citations were identified, of these 925 were excluded at title stage and 265 at abstract stage. We assessed 28 primary studies and 21 reviews for potentially relevant studies. We did not find any additional citations within the reviews. Of the primary studies, 12 were excluded due to not being conducted within a GDM population (however, some included a limited number of women with GDM as ‘high-risk’ individuals but results could not be separated). A further three articles were excluded due to being conducted solely in pregnancy, not targeting weight loss/behaviour change or not reporting the results of an intervention (see Fig. 1).

In total 13 studies were included in the systematic review (Ratner et al., 2008, Cheung et al., 2011, Ferrara et al., 2011, McIntyre et al., 2012, Cheung et al., 2007, Hu et al., 2012, Kim et al., 2012, Peterson and Jovanovic, 1995, Philis-Tsimikas et al., 2014, Reinhardt et al., 2012, Shyam et al., 2013, Shek et al., 2014, Wein et al., 1999), five in the meta-analysis of anthropometric outcomes (McIntyre et al., 2012, Hu et al., 2012, Kim et al., 2012, Reinhardt et al., 2012, Shyam et al., 2013, Shek et al., 2014, Wein et al., 1999) and four in the meta-analysis of glucose outcomes (McIntyre et al., 2012, Hu et al., 2012, Kim et al., 2012, Shyam et al., 2013). Two eligible studies included in the review were found as a result of the cited reference search, while all others were identified via the database search.

### Study characteristics

Table 1 summarizes study descriptors, intervention and comparator conditions, outcomes and findings.

Of the 13 studies, ten were RCTs (Ratner et al., 2008, Cheung et al., 2011, Ferrara et al., 2011, McIntyre et al., 2012, Hu et al., 2012, Kim et al., 2012, Reinhardt et al., 2012, Shyam et al., 2013, Shek et al., 2014, Wein et al., 1999). Two studies were pre–post (Cheung et al., 2007, Philis-Tsimikas et al., 2014) and one was an RCT cross-over design (Peterson and Jovanovic, 1995). All RCTs, except (Ratner et al., 2008), adopted a two-group design. Ratner et al. (Ratner et al., 2008) reported data from women with a history of GDM from the DPP intervention, using a three-group design (i.e. lifestyle intervention, metformin and a drug–placebo control). Five studies took place in the US (Ratner et al., 2008, Ferrara et al., 2011, Kim et al., 2012, Peterson and Jovanovic, 1995, Philis-Tsimikas et al., 2014), five in Australia (Cheung et al., 2011, McIntyre et al., 2012, Cheung et al., 2007, Reinhardt et al., 2012, Wein et al., 1999), one in China (Hu et al., 2012), one in Hong Kong (Shek et al., 2014) and one in Malaysia (Shyam et al., 2013).

#### Interventions

Three study interventions targeted physical activity only, through face-to-face counselling and follow-up phone calls (Cheung et al., 2011, McIntyre et al., 2012) or a web-based pedometer intervention (Kim et al., 2012). Two targeted diet only through through face-to-face counselling (Peterson and Jovanovic, 1995) or telephone-based education (Wein et al., 1999). Eight targeted a combination of diet and physical activity (Ratner et al., 2008, Ferrara et al., 2011, Moher et al., 2001, Cheung et al., 2007, Peterson and Jovanovic, 1995, Philis-Tsimikas et al., 2014, Reinhardt et al., 2012, Shyam et al., 2013). Three studies provided information related to intervention adherence (Kim et al., 2012, Ferrara et al., 2011, Philis-Tsimikas et al., 2014).

#### Comparators

Comparison conditions were metformin and a placebo drug (Ratner et al., 2008), educational information focused on conventional dietary recommendations (Shyam et al., 2013), written educational materials (Cheung et al., 2011, Ferrara et al., 2011, McIntyre et al., 2012, Hu et al., 2012, Wein et al., 1999), and usual care/no treatment (Reinhardt et al., 2012, Shek et al., 2014). Hu et al. (Hu et al., 2012) also provided lifestyle change information via two face to face education classes at baseline and annually via phone/mail. In Wein et al. (Wein et al., 1999), the intervention group received dietary intervention, however both groups were “advised to exercise regularly” (e.g. at least 30 min, three times per week). In Peterson et al. (Peterson and Jovanovic, 1995) participants acted as their own comparator condition with a change in dietary prescription (from 40% to 55% or 55% to 40% of carbohydrate content) at the mid-point (6-weeks) of the intervention.

#### Recruitment

12 studies provided information on recruitment methods used (see Table 1). The majority of studies recruited participants from hospital clinic settings (Cheung et al., 2011, Ferrara et al., 2011, Kim et al., 2012, Peterson and Jovanovic, 1995, Philis-Tsimikas et al., 2014, Reinhardt et al., 2012, Shyam et al., 2013, Shek et al., 2014, Wein et al., 1999). Recruitment ranged from 7% to 28% of all GDM clinic attendees (where information available (Cheung et al., 2011, Ferrara et al., 2011, Kim et al., 2012, Reinhardt et al., 2012)). A large number of women with GDM were contacted, with rates of successful recruitment varying between 19 and 70% (Table 1). Hu et al. (Hu et al., 2012) reported the most favourable recruitment rate using follow-up call(s) after mailing out a study letter to clinic attendees. Poorest recruitment was in Kim et al. (Kim et al., 2012), where participants had to sign up proactively by providing an email address (Kim et al., 2012). It took 10 months (Reinhardt et al., 2012) to recruit for a small study (< 50 participants) and between 2–4 years for studies with > 100 participants (Ferrara et al., 2011, Hu et al., 2012, Shek et al., 2014). However, most studies provided no details on length of time to recruit.

#### Retention

Retention rate at the last follow-up point in the included studies was generally between 80–100%(Cheung et al., 2011, Ferrara et al., 2011, McIntyre et al., 2012, Cheung et al., 2007, Hu et al., 2012, Kim et al., 2012, Reinhardt et al., 2012, Shyam et al., 2013). In two studies, the last follow-up point was at 12–13 weeks (McIntyre et al., 2012, Kim et al., 2012) and was between 6–12 months after baseline in other studies (see Table 1). Three studies reported good retention at later follow-ups (i.e. > 90%(Ratner et al., 2008; Shyam et al., 2013; Shek et al., 2014) at three years and (Wein et al., 1999) at 51 months). Limited details were provided on reasons for loss to follow-up or methods used to retain participants across most studies. Two studies reported lower retention [i.e. 77% [26] and 68% at six months and 12 weeks (Peterson and Jovanovic, 1995), respectively].

#### Methodological quality

Table 2 presents an assessment of risk of bias for each study. Overall, three of the 13 studies were rated as low risk of bias (Ratner et al., 2008, Ferrara et al., 2011, Kim et al., 2012), all used blinded outcomes assessors and provided details of how the randomization sequence was independently developed and allocation to study groups was concealed from investigators. Three studies were rated as high risk of bias, due to studies being non-controlled (Moher et al., 2001, Philis-Tsimikas et al., 2014) or randomization being known prior to baseline assessments (Reinhardt et al., 2012). Seven (54%) were unclear as key study indicators were not adequately described (Cheung et al., 2011, McIntyre et al., 2012, Hu et al., 2012, Peterson and Jovanovic, 1995, Shyam et al., 2013, Shek et al., 2014, Wein et al., 1999).

### Changes in behavioural outcomes

Eleven studies reported changes in behavioural outcomes (Ratner et al., 2008, Cheung et al., 2011, Ferrara et al., 2011, McIntyre et al., 2012, Cheung et al., 2007, Hu et al., 2012, Kim et al., 2012, Reinhardt et al., 2012, Shyam et al., 2013, Wein et al., 1999). See Table 1 for changes in physical activity, diet and sedentary behaviour.

#### Physical activity

Eleven studies reported on change in physical activity behaviour. Six studies found significant increases in physical activity among women with prior GDM after receiving lifestyle interventions targeting PA only (McIntyre et al., 2012), PA and diet (Ratner et al., 2008, Cheung et al., 2007, Hu et al., 2012, Philis-Tsimikas et al., 2014, Reinhardt et al., 2012). Only one of these studies was rated as low risk of bias (Ratner et al., 2008). Of the six studies reporting change, three were change from baseline to follow-up (Ratner et al., 2008, Cheung et al., 2007, Philis-Tsimikas et al., 2014) and three were compared to physical activity behaviour among controls (McIntyre et al., 2012, Hu et al., 2012, Reinhardt et al., 2012).

#### Sedentary time

Two studies reported on change in sedentary behaviour via self-reported sitting time (Hu et al., 2012, Reinhardt et al., 2012). Both report significant declines relative to the control group following lifestyle interventions, although the changes were small and associated with large confidence intervals. Neither study was rated as low risk of bias.

#### Diet

Six studies reported on change in dietary intake (Ferrara et al., 2011, Hu et al., 2012, Philis-Tsimikas et al., 2014, Reinhardt et al., 2012, Shyam et al., 2013, Wein et al., 1999). All found some positive effects on some dietary variables favouring the intervention group (Ferrara et al., 2011, Hu et al., 2012, Philis-Tsimikas et al., 2014, Reinhardt et al., 2012, Shyam et al., 2013, Wein et al., 1999) including one study rated as low risk of bias (Ferrara et al., 2011). In one study changes in dietary variables were from baseline (Philis-Tsimikas et al., 2014) and in three studies changes were relative to the control group (Ferrara et al., 2011, Hu et al., 2012, Reinhardt et al., 2012). One study found that both intensive and low-intensity (i.e. written) dietary advice resulted in modest improvements to diet (Wein et al., 1999). In one study (Shyam et al., 2013) both groups received different dietary interventions (i.e. focusing on low glycaemic index or conventional low-fat dietary advice) with resultant favourable changes in dietary variables.

### Changes in anthropometric outcomes

Anthropometric outcomes were reported in all 13 studies: weight in nine studies (Ratner et al., 2008, McIntyre et al., 2012, Cheung et al., 2007, Hu et al., 2012, Kim et al., 2012, Peterson and Jovanovic, 1995, Philis-Tsimikas et al., 2014, Reinhardt et al., 2012, Shyam et al., 2013); BMI in eight studies (Cheung et al., 2011, Cheung et al., 2007, Hu et al., 2012, Kim et al., 2012, Philis-Tsimikas et al., 2014, Reinhardt et al., 2012, Shyam et al., 2013, Shek et al., 2014, Wein et al., 1999); percent body fat in three studies (McIntyre et al., 2012, Hu et al., 2012, Shyam et al., 2013); waist circumference in five studies (McIntyre et al., 2012, Hu et al., 2012, Kim et al., 2012, Reinhardt et al., 2012, Shyam et al., 2013) and hip circumference in two studies (Hu et al., 2012, Kim et al., 2012). Two studies reported on proportion achieving weight loss goals (Ferrara et al., 2011, Shyam et al., 2013). Peterson et al. (Peterson and Jovanovic, 1995) and Wan Man Shek et al. (Shek et al., 2014) measured percent body fat and waist-hip ratio respectively but did not provide results.

Six studies found a significant reduction in weight, BMI, percent body fat and/or waist–hip ratio among participants taking part in the intervention group (Ratner et al., 2008, Cheung et al., 2007, Hu et al., 2012, Peterson and Jovanovic, 1995, Reinhardt et al., 2012, Shyam et al., 2013). Again only one of these studies (Ratner et al., 2008) was rated as low risk of bias. Of these, three were changes from baseline (Ratner et al., 2008, Cheung et al., 2007, Peterson and Jovanovic, 1995) and three were relative to a control group (Hu et al., 2012, Reinhardt et al., 2012, Shyam et al., 2013).

Among lifestyle interventions targeting diet and physical activity/sedentary behaviour, Ratner et al. (Ratner et al., 2008) reported average weight loss for women with a history of GDM within the lifestyle intervention group of 5 kg at six months, however this was not maintained until three years and therefore weight loss was poorer at three years compared to weight loss among women with impaired glucose tolerance without a history of GDM. Two other studies reported favourable changes compared to controls (Hu et al., 2012, Reinhardt et al., 2012).

Seven other studies found no significant effects of lifestyle interventions on anthropometric outcomes at follow-up (Cheung et al., 2011, Ferrara et al., 2011, McIntyre et al., 2012, Kim et al., 2012, Philis-Tsimikas et al., 2014, Shek et al., 2014, Wein et al., 1999). However, Wan Man Shek et al. (Shek et al., 2014) found the difference between the groups in reduction in weight and percent body fat were close to significance (p = 0.06 and p = 0.05), with heavier participants being more likely to be diagnosed with type two diabetes at 36 months follow-up). Also Ferrara et al. (Ferrara et al., 2011) found lifestyle intervention participants were more likely to reach postpartum weight loss goals, but only if they had not gained excessive gestational weight during pregnancy.

#### Meta-analysis of weight outcomes

**Fig. 2:**
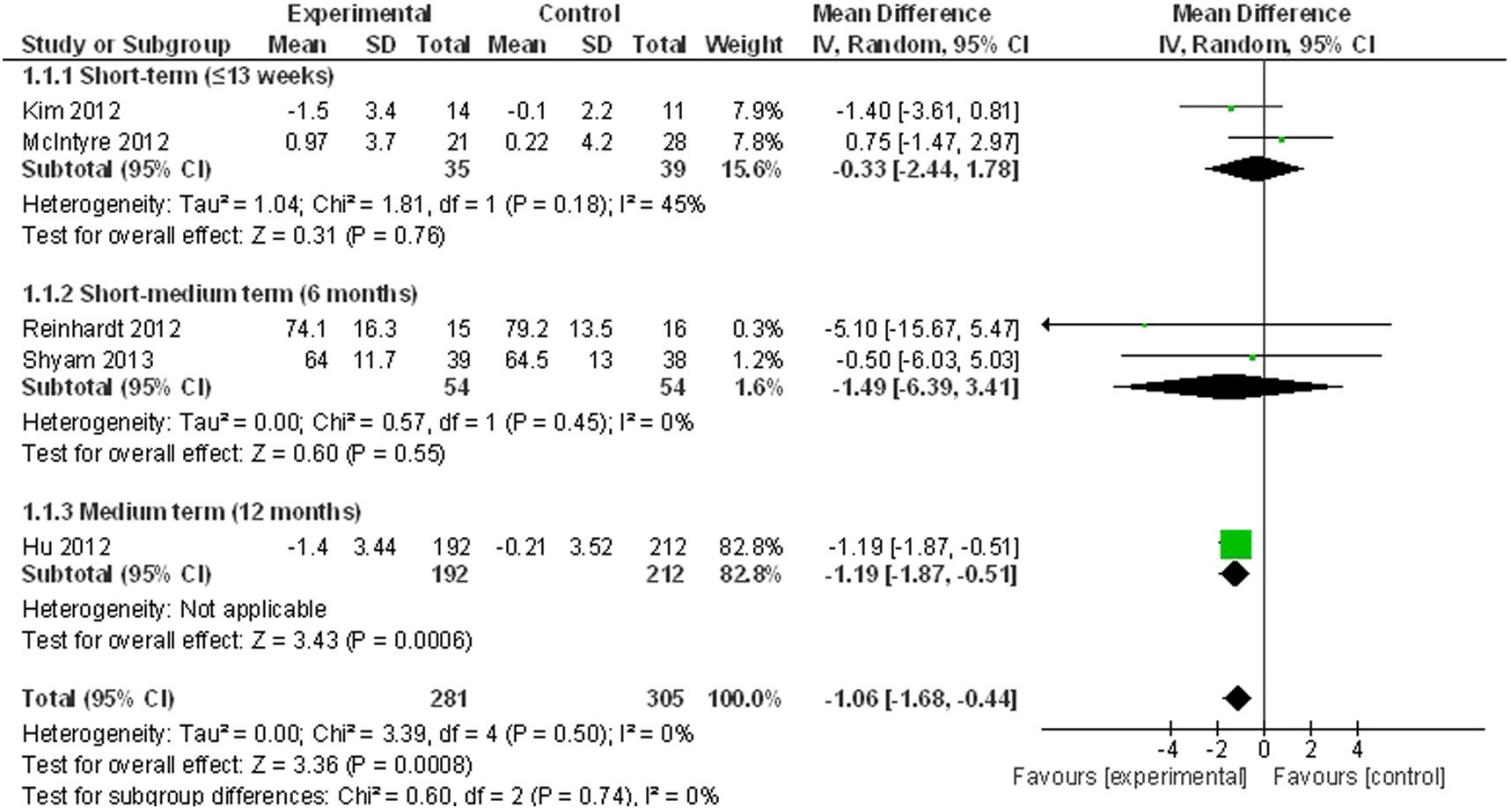
Meta-analysis of weight loss.

Lifestyle interventions resulted in a statistically significant reduction in weight (kg) based on data from five studies (McIntyre et al., 2012, Hu et al., 2012, Kim et al., 2012, Reinhardt et al., 2012, Shyam et al., 2013), see Fig. 2 (WMD = − 1.06 kg (95% CI = − 1.68, − 0.44, p < 0.01, I = 0%). However, as shown in Fig. 2, this significant effect was attributable to the reduction at 12 months follow-up in Hu et al. (Hu et al., 2012) (i.e. − 1.19 kg, 95% CI = − 1.87, − 0.51) due to the large sample size (and therefore weighting) of this trial.

### Changes in glycaemic outcomes and diabetes risk

#### Glycaemic control outcomes

Glycaemic outcomes were reported in nine studies (McIntyre et al., 2012, Cheung et al., 2007, Hu et al., 2012, Kim et al., 2012, Peterson and Jovanovic, 1995, Philis-Tsimikas et al., 2014, Shyam et al., 2013, Shek et al., 2014, Wein et al., 1999) including one study rated as low risk of bias (Kim et al., 2012). These were: HbA1c (Hu et al., 2012, Philis-Tsimikas et al., 2014), fasting insulin (McIntyre et al., 2012, Hu et al., 2012, Kim et al., 2012, Peterson and Jovanovic, 1995), fasting blood glucose (McIntyre et al., 2012, Cheung et al., 2007, Hu et al., 2012, Kim et al., 2012, Shyam et al., 2013, Wein et al., 1999), 2-hour blood glucose (Cheung et al., 2007, Hu et al., 2012, Kim et al., 2012, Shyam et al., 2013, Wein et al., 1999), and HOMA–IR (McIntyre et al., 2012, Hu et al., 2012, Shek et al., 2014). Four studies did not report any glycaemic outcomes (Ratner et al., 2008, Cheung et al., 2011, Ferrara et al., 2011, Reinhardt et al., 2012). Overall three studies reported a significant positive effect of lifestyle interventions on at least one glycaemic outcome (Hu et al., 2012, Shyam et al., 2013, Wein et al., 1999). Effects reported included a reduction in 2-hour blood glucose relative to controls among those receiving dietary interventions only (Shyam et al., 2013, Wein et al., 1999) and reduced HOMA–IR and fasting insulin relative to controls (Hu et al., 2012). In five studies, there was no effect of lifestyle interventions on glycaemic outcomes from baseline (McIntyre et al., 2012, Cheung et al., 2007, Peterson and Jovanovic, 1995) or relative to controls (McIntyre et al., 2012, Kim et al., 2012, Shek et al., 2014). In one non-controlled study there was an increase in HbA1c from baseline (Philis-Tsimikas et al., 2014).

#### Meta-analysis of glycaemic outcomes

**Fig. 3:**
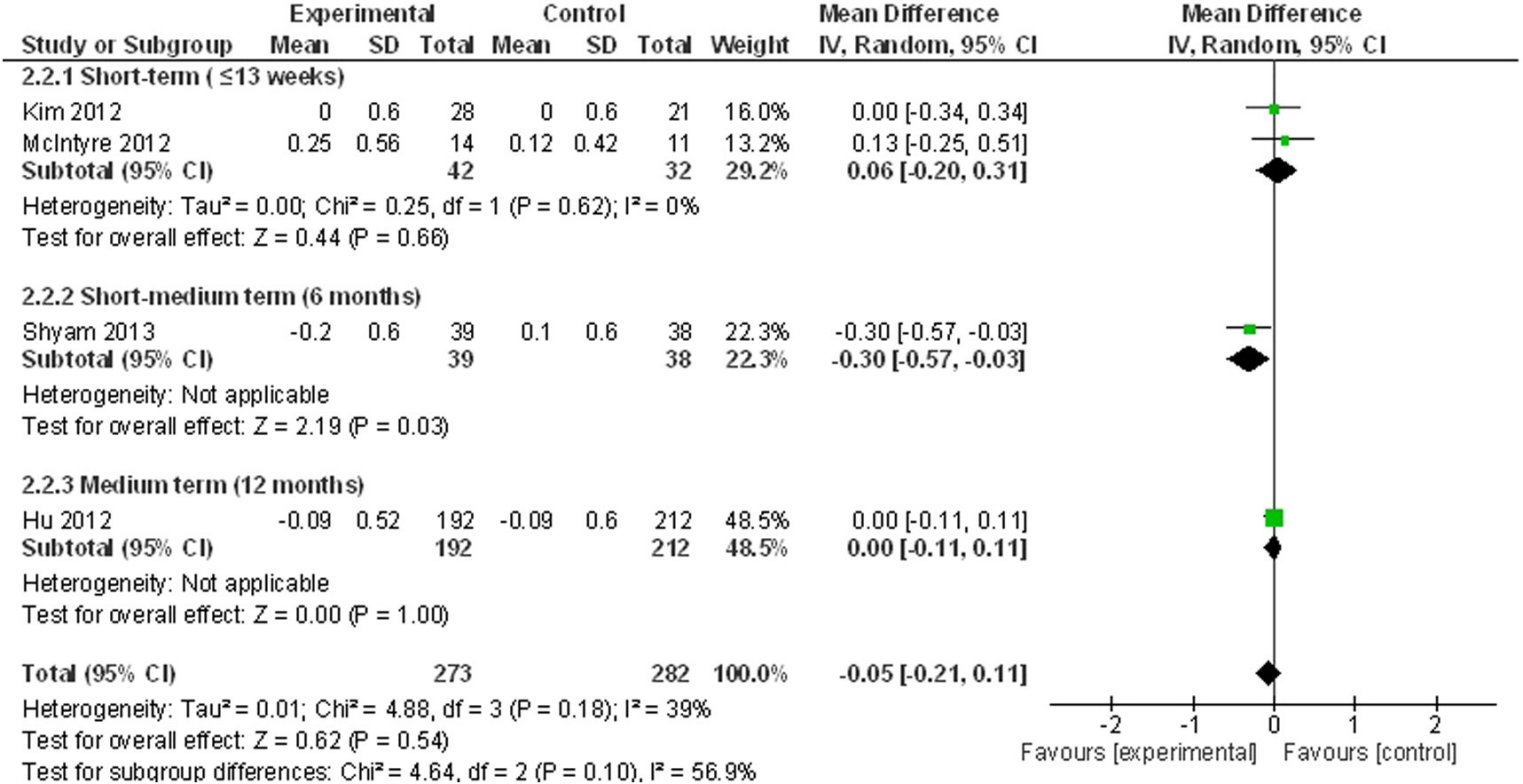
Meta-analysis of fasting blood glucose change.

Lifestyle interventions did not result in a statistically significant reduction in fasting blood glucose based on data from four studies (McIntyre et al., 2012, Hu et al., 2012, Kim et al., 2012, Shyam et al., 2013) (The WMD = − 0.05 mmol/L, 95% CI = − 0.21, 0.11, p = 0.54, I = 39, see Fig. 3).

#### Progression to type 2 diabetes

Three studies reported on progression to type 2 diabetes (Ratner et al., 2008, Shek et al., 2014, Wein et al., 1999). Findings at 36 months (Shek et al., 2014) and 51 months (Wein et al., 1999) were non-significant for rate reduction in diabetes risk. Ratner et al. (Ratner et al., 2008) reported that lifestyle intervention was equally effective at reducing the rate of diabetes progression in women with and without a history of GDM. The numbers needed to treat with lifestyle intervention was higher among GDM women compared with women without GDM. Two studies reported on progression to normoglycemia (Cheung et al., 2011, Shyam et al., 2013). Shyam et al. (Shyam et al., 2013) reported the difference in rate was non-significant at six months (see Table 1). Cheung et al. (Cheung et al., 2011) reported 63% returned to normoglycemia among the intervention group, compared to 75% among controls with no significance testing reported.

#### Other clinical outcomes

Although the objective of the review did not include extracting or reporting on other clinical outcomes three studies measured changes in blood pressure (BP) (i.e. systolic BP (Hu et al., 2012, Philis-Tsimikas et al., 2014, Shek et al., 2014), diastolic BP (Hu et al., 2012, Philis-Tsimikas et al., 2014, Shek et al., 2014)) and blood lipids (i.e. triglyceride (Hu et al., 2012, Peterson and Jovanovic, 1995, Philis-Tsimikas et al., 2014, Shek et al., 2014), serum cholesterol (Peterson and Jovanovic, 1995), HDL-cholesterol (Hu et al., 2012, Philis-Tsimikas et al., 2014, Shek et al., 2014), LDL-cholesterol (Hu et al., 2012, Philis-Tsimikas et al., 2014, Shek et al., 2014) and total cholesterol (Hu et al., 2012, Philis-Tsimikas et al., 2014)). Details of changes in these clinical outcomes following lifestyle interventions are provided in Table 1.

## Discussion

The results of this systematic review and meta-analysis suggest there is currently limited evidence from high quality studies on the effect of lifestyle interventions on behavioural, anthropometric and glycaemic outcomes among women with prior GDM.

### Study characteristics

Study quality was poor with only 3 out of the 13 studies reviewed being rated as low risk of bias. None of the studies included were conducted in Europe. The majority of research targets both diet and physical activity. Interventions are mostly delivered through face to face contact. Few studies report of intervention adherence.

Recruitment to trials within this population appears to be challenging, but trials tend to achieve high retention rates. The majority of studies recruit from hospital clinics and few provide any detail on length of time required for recruitment. More research is required which explores feasible, acceptable and effective methods of recruitment to lifestyle interventions for this group of the population. There is tentative evidence that recruitment and starting lifestyle intervention during pregnancy is beneficial (Ferrara et al., 2011). However qualitative research reports the feeling among women that the early postpartum stage is “*too early*” for considering lifestyle change (Cheung et al., 2011, Cheung et al., 2007). Later recruitment could be targeted during annual glucose monitoring (National Institute of Health and Care Excellence, 2008).

Notably, previous analyses have suggested that later diabetes risk is influenced by a variety of risk factors including diagnostic glucose levels and ethnicity with more variable results between trials for family history, BMI and insulin use (Kim et al., 2002). In general, intervention trials have not stratified for these risk factors or recently introduced categories of “overt diabetes in pregnancy” in the IADPSG and

Diabetes mellitus in pregnancy WHO. An exception is the DPP which recruited women with post partum IGT. It could be speculated that trials stratified to those most at risk might be more successful in recruitment and interventions.

### Changes in behavioural outcomes

There is minimal evidence for a change in physical activity following lifestyle intervention in women with prior GDM, with six out of eleven studies reporting favourable change. The majority of these studies were rated as high or unclear risk of bias. The exception was the DPP, which was rated as low risk of bias and reported increased physical activity relative to the control group of 150 min of moderate–vigorous activity at one year (Ratner et al., 2008). However, these changes were not sustained at three year follow up (Ratner et al., 2008). It is worth highlighting that DPP recruited women with prior GDM on average 12 years since delivery, which may not be generalizable to a population of women with prior GDM who are recruited for lifestyle intervention at early stages (i.e. during pregnancy and/or within the first few years following delivery).

Findings on change in physical activity from this review need to be interpreted with some caution as all studies measured change in self-reported physical activity and sedentary behaviour. There is evidence that self-report measures can lead to under and overestimation of participation in physical activity (Long et al., 2013). Future research should incorporate objective methods (i.e. accelerometers) of measuring physical activity and sedentary behaviour. In addition few include sedentary behaviour in their interventions (Ferrara et al., 2014, Infanti et al., 2013a, Shih et al., 2013). This is important as sedentary behaviour is increasingly recognized as an important target for improving cardio metabolic indicators of type 2 diabetes (Henson et al., 2013).

There was somewhat stronger evidence regarding change to dietary variables. One high-quality study found a reduction in percentage calories from fat among women engaged in an adapted version of the DPP intervention (Ferrara et al., 2011). However, timing of recruitment may be important. Evidence from this review and other studies (Infanti et al., 2013b) suggests women who successfully adopt lifestyle changes during pregnancy may be more likely to return for preventative support (Infanti et al., 2013b) and be more successful at maintaining dietary change postpartum (Ferrara et al., 2011). A number of studies in this review measured lifestyle intervention effects on dietary change; those not recruiting during pregnancy were also favourable, though these were of low quality or in unique populations (Philis-Tsimikas et al., 2014, Reinhardt et al., 2012, Shyam et al., 2013, Wein et al., 1999). Therefore, evidence for a positive impact of lifestyle interventions in women with prior GDM on dietary variables postpartum remains tentative.

Future research should also give consideration to the wider socio-economic, social and cultural environment in which women with prior GDM live, for example, there is evidence that inclusion of partners is important for changing physical activity and dietary behaviours among women with young children (Fjeldsoe et al., 2010 May, Miller et al., 2002) and is desired by women with prior GDM (Dasgupta et al., 2013).

### Changes in anthropometric outcomes

There was limited evidence in this review for significant changes in anthropometric outcomes following lifestyle interventions among women with prior GDM. Although the meta-analyses for weight and BMI were statistically significant, the magnitude of change would not be considered clinically significant (National Institute of Health and Excellence, 2010); furthermore, one trial of unclear quality, conducted in a Chinese population (Hu et al., 2012) was responsible for the small effect size found. The exception to this was in the DPP study (Ratner et al., 2008) which found significant weight loss in the first year following intensive lifestyle intervention among women with prior GDM, however this was not maintained at later stages and the population may not be generalizable, as discussed previously. Another high quality study found that an adapted DPP intervention promoted weight loss at 12 months, but only among women who successfully avoided excessive gestational weight gain during pregnancy and who received intensive lifestyle intervention immediately following GDM diagnosis (Ferrara et al., 2011). It may be that women who more successfully adopt lifestyle changes during pregnancy feel more motivated, self-efficacious and supported, helping them to maintain behavioural changes into postpartum.

Results from the DPP trial have shown maintained weight loss to be the main predictor of risk reduction in type 2 diabetes prevention in the general population with IGT (Knowler et al., 2009). Therefore, it seems pertinent to focus on developing and testing lifestyle interventions that can produce successful long-term weight reduction among women with prior GDM. The present review found no high-quality studies reporting favourable long-term weight outcomes in this group, other than the subset of women from the DPP (Ratner et al., 2008). In the general obese population long-term (≥ 12 months) weight loss has been shown following behavioural interventions focusing on both diet and physical activity change (Dombrowski et al., n.d.). Weight-loss medication improved the magnitude of weight reduction. On the one hand, among postpartum populations, dietary change alone is considered as effective for weight-loss as dietary change in combination with physical activity (Amorim Adegboye and Linne, 2013). On the other hand, physical activity and sedentary behaviour change are important, particularly as women with prior GDM are at high future high risk of cardiovascular disease (Carr et al., 2006). Physical activity is considered the most important modifiable risk factor for preventing cardiovascular disease among healthy young women (Brown et al., 2014), independent of other risk factors, including BMI. This review showed that fewer studies focused on physical activity or sedentary behaviour, compared with dietary change. This may reflect preferences among women with prior GDM regarding how, when and what lifestyle changes are adopted or a greater emphasis on dietary change in interventions targeting women with GDM.

### Changes in glycaemic outcomes and diabetes risk

Trials among women with prior GDM, not including the DPP, showed no robust evidence for change in glycaemic indicators or diabetes risk reduction, despite these being important health outcomes. However, with a few exceptions, trials did not appear to have been adequately powered or include long enough follow-ups to demonstrate change in diabetes risk reduction.

## Summary

There is consensus that prevention of type 2 diabetes should be prioritized through lifestyle interventions (National Institute of Health and Care Excellence, 2008, Buchanan et al., 2012, England et al., 2009). The recent diagnostic criteria for classification of GDM proposed by the International Association of Diabetes in Pregnancy Study Group (IADPSG) (Coustan et al., 2010) offers opportunity for early lifestyle intervention and future prevention of Type 2 diabetes and other disease over the lifespan. This review shows that we currently lack an evidence base from methodologically robust trials for how to effectively promote lifestyle change among women with prior GDM. There is evidence quality of methodology is improving with future study protocols providing more detailed information and being methodologically more robust (Ferrara et al., 2014, Infanti et al., 2013a, Shih et al., 2013, Berry et al., OCT 10 2013). Recruitment to trials and adopting lifestyle changes appear challenging in this group. Further research is urgently required to explore feasible, acceptable and effective lifestyle interventions for this target group of the population.

## Author contribution

A.S.G. contributed to conception and design of the study, led on data analysis, wrote the initial draft of the paper and reviewed the final draft. A.F.K. initiated the conception and design of the study, assisted in researching the data, reviewed initial drafts of the paper and completed the final submitted paper. A.R.H. made a substantial contribution to conception and design of the study, assisted in researching the data and reviewed initial and final drafts of the paper. R.S.L. contributed to conception and design of the study and reviewed initial and final drafts of the paper.

## Conflict of interest

None of the authors of this paper report a conflict of interest in relation to the material covered in this paper.

## Figures and Tables

**Table 1:** Study characteristics, efficacy outcomes and risk of bias for included studies.

Author [ref], country	Design, aim, setting duration, follow-up	Study population	Intervention, adherence and comparator condition	Outcome measures	Efficacy for all outcomes (last follow-up point)	Bias risk
*Physical activity interventions*						
Cheung (Cheung et al., 2011), 2011 Australia	RCT Aim: accumulate 30 mins PA OR 10,000 steps/day on 5 days Setting: hospital Intervention length: one year (adoption 0–6 months then maintenance phase 6–12 months) Follow-up: one year	N = 43 Stage: 6–24 months after a GDM pregnancy Age: 36.5 years, BMI: 27.2 kg/m^2^, Parity: NG, Ethnicity: NG, Exclusions: > 4 years since GDM diagnosis, overt diabetes, pregnancy, already active^a^ Recruitment rate: 19% of women completing a GDM survey, 7% of GDM clinic attendees Retention rate: 86%, control group 6-month courtesy call to improve retention	Intervention: face-to-face 1 hour counselling session then goal-directed phone calls at 2, 6 and 10 weeks, then 26 and 34 weeks. Pedometers for setting and monitoring goals. 7 postcards with messages to reinforce change. Staff: “*trained counsellor*” Adherence: NG Comparator: written lifestyle advice and a 6-month courtesy call.	PA: AWAS and Yamax Digiwalker pedometer Anthropometric: BMI (kg/m^2^) Diabetes progression: % with T2DM, IGT and normal BG at follow-up	30.8%) (4/13) in the intervention group met the PA target of 10,000 steps/day on 5 or more days/week, compared to 17.6% (3/17) of control group (p = 0.34) and 70.0% (14 / 20) versus 57.9% (11 / 19) achieved 150 min/week of moderate-intensity PA (p = 0.51) No differences between the groups on BMI (p = 0.14)	Unclear
McIntyre (McIntyre et al., 2012), 2012 Australia	RCT Aim: accumulate 150 mins/week planned PA Setting: hospital Intervention: 12 weeks Follow-up: 12 weeks (18 weeks PP)	N = 28 Stage: approached between delivery and 6 weeks postpartum Age: 32.8 years, BMI: 30.5 kg/m^2^, parity: > 60% more than one child Ethnicity: NG, Exclusions: NG Recruitment rate: 60% of women declined participation as was “*too early*” Retention rate: 89%	Intervention: initial face-to-face consultation for initiation followed by weekly (4 weeks) then bi-weekly (8 weeks) calls for maintenance SCT mentioned. Staff: exercise physiologist Adherence: NG Comparator: printed leaflet	PA: AWAS Anthropometric: weight (kg) and %bodyfat measured by bioelectrical impedance and waist circumference (cm) Glycaemic: FBG (mmol/L), fasting insulin (μIU/L), HOMA–IR	Intervention group increased PA by 60 mins/week, among controls 0 min/week change (NS difference between the groups, p = 0.23) % of participants increasing PA of > 60 mins/week higher among the intervention group (67% compared to 31% among controls) Fewer than half in both groups met their PA goal (150 mins/week) at follow-up Report no change on weight, anthropometric measures or glycaemic measure in either group at follow-up.	Unclear
Kim (Kim et al., 2012), 2012 US	RCT Aim: weekly stepgoals were reset based on previous week activity, never exceeding 10,000 steps/day Setting: hospital Intervention: 12 weeks Follow-up: 13 weeks	N = 49 Stage: intervention 20 months, control 14 months since GDM delivery Age: 35.7 years, BMI: 29.9 kg/m^2,^ Parity: NG, Ethnicity: > 70% non-Hispanic white, exclusions: pregnant or PA/week > 150 mins. Recruitment rate: 7% of GDM clinic attendees registered for study, 37% of these attended baseline visit Retention rate: 86%	Intervention: online delivery of a pedometer programme. Weekly stepcount recorded. Interaction via message boards, email/text feedback. Based on self-regulation, risk perception. Staff: NA Adherence: participants uploaded weekly step data 1.6 times. Comparator: usual care	PA: questionnaire and pedometer readings Anthropometric: weight (kg), waist and hip cir (cm) and BMI (kg/m^2^) Glycaemic: FBG (mmol/L), fasting insulin (mmol/L), 2-hour BG (mmol/L)	No between groups differences on proportion achieving > 60 mins/week of total, mild, moderate or vigorous PA. Small increase in stepcount among the intervention group of 543 (+/− 2074) steps/week NS between groups differences on all weight and anthropometric measures (all p > 0.10) NS between groups differences on all glycaemic measures (all p ≥ 0.10)	Low

*Diet interventions*						
Peterson (Peterson and Jovanovic, 1995), 1995 US	RCT cross-over design Aim: daily caloric prescription of 16.5 kcal/kg weight (1500 kcal/day), target weight loss of 1–2 lbs per week Setting: hospital Intervention length: 12 weeks Follow-up: 6 weeks and 12 weeks	N = 25 Stage: GDM pregnancy 1–4 years prior to recruitment Age: 34.1 years %body fat: 26.8%, parity: NG Ethnicity: NG Exclusions: NG Recruitment rate: NG Retention rate: 76% at six weeks, 68% at 12 weeks	Intervention: face-to-face weekly/bi-weekly. Dietary prescription (40% or 55% CBH) with supplements Participants maintained food diary. No behaviour change theory. Staff: NG Adherence: 32% completed protocol at 12 weeks Comparator: cross over	Anthropometric: weight and %bodyfat Glycaemic: serum fasting insulin (mU/L) Other clinical: triglycerides (mg/dl), serum cholesterol (mg/dl)	Weight loss occurred in both groups at 6 weeks (p ≤ 0.03). with further weight loss at 12 weeks NS (p-value unreported) Anthropometric measures unreported No changes in serum fasting insulin at follow-up No changes in other clinical measures at follow-up	Unclear
Wein (Wein et al., 1999), 1999 Australia	RCT Aim: “*compliance with the diet and exercise recommended at the time of diagnosis of IGT*.” Setting: hospital Intervention length: up to 6 years Follow-up: annually up to 6 years (average 51 months)	N = 200 Stage: at annual/bi-annual glucose monitoring following GDM pregnancy. All met criteria for IGT Age: 38.7 yrs, BMI: 25.3 kg/m^2,^ Parity: NG, Ethnicity: NG. Exclusions: not able to understand English Recruitment rate: NG, all women diagnosed during 3 year period. Retention rate: 96% at 51 months	Intervention: initial face-to-face contact then calls every three months. Advised to exercise regularly. No behaviour change theory. Staff: dietician Adherence: NG Comparator: “*routine*” dietary advice sheet given as part of usual care and advised to exercise (in line with intervention group)	PA & diet: questionnaire Anthropometric: BMI (kg/m^2^) Glycaemic: FBG (mmol/L), 1-hour BG (mmol/L), 2 hour BG (mmol/L), Annual incidence rate for T2DM	Increase in diet score in both group. No change in mean exercise score in either group from baseline. Increase in BMI, FBG & 1-hour BG from baseline to last follow-up point in both groups (p-value NG). Reduction in 2-hour BG (intervention) & increase (controls) (p < 0.02). Incident rate ratio between the groups of 0.83 was NS (p = 0.05, 95% CI 0.47–1.48)	Unclear

*Physical activity & diet interventions*						
Ratner (Ratner et al., 2008), 2008 US	RCT (3-group design) Aim: lose > 7% body weight through healthy eating (reduction in dietary fat to < 25% of total calories) and 150 mins/week moderate PA. Setting: healthcare, employers, social groups and mass media Intervention: initially 24 weeks with continuous follow-up support/maintenance Follow-up: 3 years	N = 350 Stage: women with IGT and GDM pregnancy (average of 12 years since delivery of 1st GDM pregnancy) Age^d^: 43 years, BMI^d^: 34.2 kg/m^2^, Parity^d^: 2.6 live births, Ethnicity^d^: 54% Caucasian Exclusions: in line with DPP^e^ Recruitment rate: NA Retention rate: 93%^e^ (at 2.8 years follow-up)	Intervention: DPP lifestyle intervention using curriculum based on modification concepts from behavioural theory Individual 30 min session then calls (16 sessions in the first 24 weeks then monthly contact). Weekly group exercise classes and after 24 weeks group education classes quarterly. Staff: lifestyle behaviour case manager Adherence: NG Comparator: 1) metformin intervention and 2) placebo drug (adherence assessed with pill counts)	PA: Modifiable activity questionnaire Diet: FFQ Anthropometric: weight (kg) and BMI (kg/m^2^) Diabetes progression: Cumulative incidence of T2DM at follow-up (per 100 person years) and numbers needed to treat	Increase in METhours/week in lifestyle intervention group compared with baseline (not sustained at 3 years). Change in dietary measures not reported. Weight loss at 6 months in the lifestyle group was 5.1 kg (not sustained at year 3). Weight loss poorer in GDM women than no-GDM women (p = 0.02). Rate of risk reduction for lifestyle (53.4%) and metformin (49%) similar compared with placebo on incidence rate (per 100 person-years) of progression to T2DM in non-GDM women but in GDM women metformin was more effective (50%) compared with lifestyle intervention (14%).	Low
Shyam (Shyam et al., 2013), 2013 Malaysia	RCT Aim: adopt a low glycaemic index (LGI) diet to achieve and maintain reduction in body weight of 5–7% (only if BMI > 23). Also encouraged to be physically active for 30 min (at least 5 times/week) Setting: hospital Intervention: 6 months Follow-up: 3 and 6 months	N = 77 Stage: at least two months post GDM delivery. Age: 31.5 years, BMI: 26.0 kg/m^2,^ Parity: 2 children, Ethnicity: NG, Exclusions: health complications, usage of drugs affecting body weight/glucose control. Recruitment rate: 35% declined, 54% of eligible women recruited (41% of those approached) Retention rate: 81%, regular contacts to improve retention (withdrawal *too busy*, lack of support, pregnancy, etc.)	Intervention: 1 face-to-face session with meal plans to follow at home. Also encouraged to be physically active for 30 min (at least 5 times/week) Up to 2 electronic (SMS—text/email) contacts/month thereafter. Individualised energy prescription capped at 1800 kcal/day. Staff: research nutritionist Adherence: NG Comparator: educational information similar to LGI group but focus on weight loss via conventional dietary recommendations	PA: IPAQ Diet: 3-day diary Anthropometric: weight (kg), BMI (kg/m^2^) WHR Glycaemic: FBG (mmol/L), 2-hr BG (mmol/L) Progression: conversion rate to normoglycemia	Difference between groups favouring the LGI intervention on dietary fibre intake, glycaemic index, glycaemic load, % protein intake and % carbohydrates intake Difference between groups favouring CHDR intervention on fat intake and % of calories from fat at 6 months. No differences in total calorie intake or median METmins between the groups. Proportion achieving 5% weight loss significantly better in the LGI group (33%) compared with 8% in the CHDR group. Changes in weight, BMI and WHR in the LGI group from baseline to 6-months follow-up (p < 0.02). No changes in the CHDR group. NS differences between the groups on FBG. LGI group maintained 2-hour BG levels with increases in the CHDR group. No differences in conversion rate from dysglycaemia to normoglycemaia (LGI 64% compared to 38% in the CHDR group, p = 0.38)	Unclear
Ferrara (Ferrara et al., 2011), 2011 US	RCT Aim^b^: postpartum, Targets: reach prepregnancy weight or lose 5% of prepregnancy weight within 12 months if BMI ≥ 25, consume 25% or less total cals/day from fat, 150 mins/week PA, exclusively breastfeed for 6 months Setting: hospital Intervention length: 12 months Follow-up: 6 weeks, 7 and 12 months PP	N = 197 Stage: during pregnancy, following GDM diagnosis Age: 77% > 30 years, BMI: intervention 55% > 30 kg/m^2^, Parity: NG, Ethnicity: > 50% Asian/Pacific islander Exclusions: high-risk pregnancy, diabetic retinopathy, thyroid disease. Recruitment rate: 46% of all GDM pregnancies contactable over 2.5 years, 84% of those contacted agreed to participate Retention rate: 80%	Intervention: prenatal face-to-face 1 hour dietary counselling session and 2 phone calls. Encourage to accumulate 150 mins/week of PA. One 1 hour breastfeeding counselling session then 1–4 calls in first 6 weeks PP. After six weeks PP 16 sessions (2 in-person) and 6–16 calls then 3 maintenance calls. Adapted DPP informed by SCT and transtheoretical model. Staff: dietician and lactation consultant Adherence: 79% ≥ 2 prenatal sessions, 74% completed 8 postpartum sessions (average 9.4). Median of 3 self-monitoring dairies returned (31% ≥ 6). Comparator: printed educational.	PA: 7-DAY PAR Diet: 12item FFQ Breastfeeding: % breastfeeding at 6 months PP Anthropometric: BMI (kg/m^2^) and % achieving weight loss goal	No difference between the groups in MVPA (p = 0.91) Reduction in %calories from fat favouring the intervention group (p < 0.01) No difference between the groups on breastfeeding (63% in the intervention group, 48% among controls, p = 0.09) 38% of intervention participants and 21% of control participants achieved weight loss goals PP (No difference between the groups, p = 0.07) Among women not exceeding GWG guidelines % reaching weight loss goals PP higher among intervention group (p = 0.04)	Low
Cheung (Cheung et al., 2007), 2007 Australia	Pre–post Aim: accumulate 30 mins/day PA on most days, reduce saturated and total fat intake, increase polyunsaturated fat. Low glycaemic index 5% reduction in body weight OR reach BMI < 25 Setting: hospital Intervention length: one year Follow-up: one year	N = 25 Stage: 6–24 months after a GDM pregnancy Age: NG, BMI: Average 29.9 kg/m^2^, Parity: NG, Ethnicity: NG Exclusions: NG Recruitment rate: NG Retention rate: 80%	Intervention: weekly face-to-face classes plus 40–50 mins of supervised exercise and pedometers, newsletters and telephone contacts used. Free childcare provided. No theory mentioned. Staff: NG for group classes, participants met with a dietician every three months Adherence: 44% attended irregularly (no definition) or dropped out Comparator: NA	PA: active Australia Questionnaire Anthropometric: Weight (kg) and BMI (kg/m^2^) Glycaemic: FBG (mmol/L), 2-hour BG (mmol/L)	Median walking increased (15 to 105 mins/week, p < 0.01). Vigorous activity and LTPA increases were NS. Reduction in weight and BMI: 29.9 to 29.1 (p ≤ 0.04) NS changes in glycaemic outcomes	High
Hu (Hu et al., 2012), 2012 China	RCT Aim: accumulate 30 mins/day moderate or vigorous PA on 7 days/week, fat < 30%, saturated fat < 10%, carbohydrate 55–65%, fibre intake 20–30 g/day, reduction of 5–10% body weight (BMI ≥ 24) Setting: hospital Intervention: two years (year 2 maintenance) Follow-up: one year	N = 1180^c^ Stage: NG Age: 32 years, BMI: 23.9 kg/m^2,^ Parity: NG, Ethnicity: all Chinese Exclusions: < 20 or ≥ 50 years old, taking medications altering blood glucose, chronic disease or pregnant/intention to become pregnant Recruitment rate: 36% of all GDM pregnancies over four years contacted. 70% of those contacted agreed to participate. In total recruited 25% of the GDM clinic sample Retention rate: 92% for first 444 participants (ongoing trial)	Intervention: two-week with two face-to-face education classes. Then personalised dietary/PA advice and 5-day meal plan, exercise goals, delivered face-to-face and calls for one year (minimum 5 contacts in year 1) with goal-monitoring. Then 2 face-to-face contacts and 2 calls in year two. No theory mentioned. Staff: dietician Adherence: NG Comparator: took part in initial 2 week education session then yearly oral/written information	PA: self reported Diet: 3-day 24-hour food diary, Anthropometric: weight (kg), waist and hip circumference, BMI and %bodyfat measured by bioelectrical impedance, Glycaemic: FBG, fasting insulin, 2-hour BG, HOMA–IR, HbA1c Other clinical: BP, lipids	% of participants increasing LTPA higher among the intervention group (59% vs 27%). Reduction in sitting time in intervention group and increase among controls, No differences in most dietary outcomes with exception of intervention group reporting increased fibre consumption. Between groups differences on all weight and anthropometric outcomes favouring the intervention, except NS difference in hip circumference. Between groups differences on reduction in fasting insulin and HOMA–IR favouring the intervention. s	Unclear
Philis-Tsimikas (Philis-Tsimikas et al., 2014), 2014 US	Pre–post study Aim: non-specific weekly healthy lifestyle goals targeting diet and PA Setting: hospital clinic and community health centre Intervention: three months (then unspecified support/maintenance) Follow-up: 3 and 6 months	N = 84 Stage: GDM pregnancy in previous three years (73% were < 2 years since delivery) Age: 32 years, all 18–45 years BMI: 29.0 kg/m^2^, Parity: NG Ethnicity: all low-income Latino Exclusions: NG Recruitment rate: 34% declined participation, 50% of eligible women recruited (32% of approached sample) Retention rate: 77%, withdrawal due to barriers or return to Mexico	Intervention: 8 weekly 2 hour culturally sensitive group education sessions (5–12 participants) with 15–20 min PA. Adapted-DPP (SCT). Staff: peer educators (training and support from a multidisciplinary health professional team). Adherence: attendance averaged 6/8 classes, 90% ≥ 4 classes, 17% attended 8 classes Comparator: NA	PA: Rapid PA assessment Diet: diet screening tool Anthropometric: weight (lbs) and BMI (kg/m^2^) Glycaemic: HbA1c Other clinical: BP, triglycerides, HDL-C, LDL-C, total cholesterol	Increase in the proportion of participants who were active for ≥ 30 mins 5/week (from 52% to 69%, p < 0.05). Increase in the proportion of participants doing any strength/flexibility training (from 18% to 64%, p < 0.01) Decrease in the intake of dietary fat (as % total calories) from 34% to 31%, (p < 0.01) NS change on weight and BMI (p > 0.20) Increase in HbA1c over time (p < 0.05) Decrease in DBP, triglycerides, LDL-C and total cholesterol over time (all p < 0.05). NS change HDL-C and SBP (all p > 0.20)	High
Reinhardt (Reinhardt et al., 2012), 2012 Australia	RCT Aim: non-specific weekly healthy lifestyle goals targeting diet and PA Setting: hospital clinic Intervention: 6 months Follow-up: 6 months	N = 38 Stage: 6 weeks after delivery Age: 32.5 years, BMI: intervention 29.0 kg/m^2,^ Parity: 2.3 children, Ethnicity: NG, only recruited rural women Exclusions: no access to a phone, medical contraindications Recruitment rate: 17% of women using the GDM clinic over 10 months Retention rate: 84%, weekly follow-up calls to improve questionnaire data collection	Intervention: 10 calls: weekly (5 weeks) then monthly (5 months). Self-help booklet. Reviewed behaviours/barriers at calls. Offered 2 exercise classes/week. Based on determinants (benefits, perceived barriers, SS etc.) using MI. Staff: diabetes educators, Adherence: NG, pre-arranged phone sessions Comparator: usual care	PA: IPAQ Diet: Cancer Council FFQ Anthropometric: weight, BMI and waist circumference	Difference between groups favouring the intervention on LTPA Increase in total PA favouring the intervention. Decrease in sitting time favouring the intervention. Decrease in fat, carbohydrate intake & glycaemic load favouring the intervention. No difference in saturated fat or fibre intake. No difference in weight/BMI. Decrease in waist circumference favouring the intervention.	High
Wan Man Shek (Shek et al., 2014), 2014 Hong Kong	RCT Aim: non-specific healthy lifestyle goals targeting diet and PA Setting: hospital Intervention length: up to 36 months Follow-up: every three months until 36 months	N = 450 Stage: 6–8 weeks since delivery of GDM pregnancy and meeting criteria for IGT Age: 39 years, BMI: 24.5 kg/m^2^, Parity: 1.7 children, Ethnicity: Chinese Exclusions: no communication, used insulin during pregnancy Recruitment rate: recruitment over 3 years from GDM clinic, rate NG Retention rate: 94%	Intervention: one face-to-face lifestyle consultation repeated every three months up to 36 months. Individualised calculation of calorie intake with monitoring of food and PA by diaries (checked at visits). No behavioural theory mentioned. Staff: dietician (first visit) then research nurse Adherence: NG Comparator: no treatment	Anthropometric: Weight, BMI, WHR, %body fat Glycaemic: FBG, 2 h BG, fasting insulin, HOMA index T2DM progression: cumulative T2DM rate. Other Clinical: BP, lipids	Reduction in %body fat, triglycerides, SBP at last follow-up in the intervention. No difference in BMI & WHR at last follow-up in the intervention group from baseline. No differences in conversion rate to T2DM. Among subgroup of women > 40 years old a reduction in conversion rate favoured the intervention group. No difference at last follow-up among intervention group on all glycaemic measures or other clinical measures.	Unclear

7-DAY PAR, Seven Day Physical Activity Recall; ADA, American Dietary Association; AWAS, Australian Women's Activity Survey; BMI, body mass index; CI, confidence interval; CNNHS, China National Nutrition and Health Survey; BP, blood pressure DBP, diastolic blood pressure; SBP, systolic blood pressure, DPP, Diabetes Prevention Program; FBG, fasting blood glucose, FFQ, food frequency questionnaire; GDM, gestational diabetes mellitus; GWG, gestational weight gain; HDL-C, high-density lipoprotein; IGT, impaired glucose tolerance, IOM, Institute of Medicine; IPAQ, International Physical Activity Questionnaire; LDL-C, low-density lipoprotein; LTPA, leisure-time physical activity; mins, minutes; motivational interviewing, MI; MVPA, moderate–vigorous physical activity; NA, not applicable; NG, not given; NS, non-statistically significant; PA, physical activity; PP, postpartum; RCT, randomised controlled trial; SCT, socio-cognitive theory; T2DM, type 2 diabetes mellitus; WHR, waist–hip ratio.

Note.

All studies excluded women with current diagnosis of type 2 diabetes mellitus.

a Determined by questionnaire and pedometer readings > 62,000 steps/week.

b Prenatal targets: GWG in line with IOM guidelines (or not greater than 11.4 kg), follow the ADA diet (i.e. low fat, low glycaemic index); engage in 150 mins/week moderate PA.

c Number enrolled in the study, this paper reports data for the first 394 participants who completed one year data assessments at the end of November 2011.

d Participants were women with a history of GDM and were part of the larger DPP trial and were not comparable to women with at least one live birth without a history of GDM in terms of age but were comparable on ethnicity, BMI and number of live births.

e Complete list of exclusions and study retention rate for the DPP is provided by Knowler et al., (2009).

**Table 2:** Risk of bias among included studies.

Author(s), year	Sequence generation	Concealed allocation	Outcome assessment	Loss-to follow-up	Missing data handling	Overall risk of bias	Rationale for overall risk
Cheung, 2007 (Cheung et al., 2007)	N/A	N/A	U^a^	A	A	High	Pre–post study lacking randomly allocated control
Cheung, 2011 (Cheung et al., 2011)	U	U	U^a^	A	N	Unclear	Unclear how participants were allocated and randomised and extent of concealment. Low number included in the analysis.
Ferrara, 2011 (Ferrara et al., 2011)	A	A	A	A	A	Low	All quality criteria adequate.
Hu, 2012 (Hu et al., 2012)	U	U	U^a^	A^b^	A^b^	Unclear	Early results suggest low drop-out from study, randomisation after all baseline assessments completed but unclear whether allocation was concealed.
Kim 2012 (Kim et al., 2012)	A	A	A^c^	A	A	Low	All quality criteria adequate.
McIntyre, 2012 (McIntyre et al., 2012)	U	U	U^a^	A	N	Unclear	Unclear how participants were allocated and randomised and extent of concealment
Peterson, 1995 (Peterson and Jovanovic, 1995)	U	U	U^d^	N^d^	U	Unclear	Main study indicators unclear
Philis-Tsimikas, 2014 (Philis-Tsimikas et al., 2014)	N/A	N/A	U^a^	N	N	High	Pre–post study lacking randomly allocated control
Ratner, 2008 (Ratner et al., 2008)	A	A	A^e^	A	A	Low	All quality criteria adequate with low loss-to-follow-up and ITT used.
Reinhardt et al, 2012 (Reinhardt et al., 2012)	U	N	N	A	A	High	Randomisation to groups known by researcher (and possibly participants) prior to baseline assessments
Shyam et al, 2013 (Shyam et al., 2013)	A	U	U^a^	A	A	Unclear	Unclear whether allocation to groups was adequately concealed and whether anthropometric and/or dietary outcome^f^ assessors were blinded to group.
Shek et al, 2014 (Shek et al., 2014)	A	U	A	A	U	Unclear	Unclear whether allocation to trial arms was concealed prior to baseline assessments or how loss-to-follow up data was imputed.^g^
Wein, 1999 (Wein et al., 1999)	U	U	U^a^	A	A	Unclear	Unclear how participants were allocated and randomised and extent of concealment

A, adequate; ITT, intention-to-treat; N, not adequate; U, unclear, N/A, not applicable

Note.

a Lack of blinding likely to affect self-report behavioural measures/waist or hip measurements but not weight/objective physical activity outcomes.

b 92% retention at one year for the first 444 participants recruited into the study.

c Limited information on how behavioural or weight outcomes were assessed, mentions anthropometric testing and online survey only.

d Unclear how weight, waist and hip measurements taken, drop-out at 6 weeks 24% and at 12 weeks 32%.

e Double blinded for drug/placebo groups, investigators masked to treatment assignment unless diabetes diagnosis confirmed.

f Other possible threat to validity is the use of multiple tests for weight, anthropometry and diet measure.

g States ITT for diabetic outcomes but not clear for weight outcomes; there was a low loss to follow-up rate, with a further small proportion becoming pregnant.
